# Effect of Regional Anesthesia on Intraoperative Hyperkalemia in Patients With Chronic Kidney Disease: Two Case Reports

**DOI:** 10.7759/cureus.74272

**Published:** 2024-11-22

**Authors:** Shunsuke Oura, Marie Okada, Ryo Miyashita

**Affiliations:** 1 Anesthesiology, Sapporo Higashi Tokushukai Hospital, Sapporo, JPN; 2 Anesthesiology, Obihiro-Kosei General Hospital, Obihiro, JPN

**Keywords:** acute kidney injury, chronic kidney disease, general anesthesia, hyperkalemia, regional anesthesia

## Abstract

Intraoperative hyperkalemia is particularly common in patients with chronic kidney disease (CKD). We report two cases of intraoperative hyperkalemia occurring under general anesthesia, while potassium levels remained stable with regional anesthesia alone. Case 1 involved a 69-year-old male with CKD who underwent total thyroidectomy under general anesthesia and developed intraoperative hyperkalemia, requiring glucose-insulin (GI) therapy. The same patient, however, maintained stable potassium levels during transurethral resection of a bladder tumor performed with spinal anesthesia. Case 2 involved a 72-year-old male with rheumatoid arthritis who underwent artificial joint replacement and tendon transplantation of his finger under a combination of general and regional anesthesia. He developed intraoperative hyperkalemia requiring GI, but stable potassium levels were maintained during bilateral total knee arthroplasty under epidural and spinal anesthesia. Our cases highlight the potential of regional anesthesia to reduce the risk of intraoperative hyperkalemia, particularly in patients with CKD.

## Introduction

Renal insufficiency reduces the glomerular filtration rate and impairs potassium excretion, which can lead to perioperative serum hyperkalemia [1]. Previous studies suggest that regional anesthesia may protect renal function and mitigate the deterioration of blood gas parameters, including intraoperative hyperkalemia [2]. This renal protective effect could help reduce the incidence of postoperative acute kidney injury [2,3]. We report two cases of intraoperative hyperkalemia requiring glucose-insulin (GI) therapy during elective surgery under general anesthesia, while no hyperkalemia was observed with regional anesthesia alone in the same patients. The potential mechanisms behind the renoprotective effects of regional anesthesia are also reviewed.

## Case presentation

Case 1

A 69-year-old male (height: 175 cm; weight: 86 kg) with Graves’ disease was scheduled for a total thyroidectomy. His medical history included chronic kidney disease (CKD) grade 4 (serum creatinine (sCr) level: 3.19 mg/dL; estimated glomerular filtration rate (eGFR): 16.18 mL/min/1.73m²), atrial fibrillation, and hypertension. He also had a left solitary functioning kidney due to hydronephrosis. In the operating room, awake tracheal intubation was performed using 200 mg of fentanyl due to tracheal deviation and stenosis attributed to a giant goiter. Following intubation, general anesthesia was induced using 100 mg of propofol and 50 mg of rocuronium. In addition to standard monitors, continuous arterial pressure was monitored via the radial artery. Anesthesia was maintained with a mixture of 35-50% oxygen, air, and 1.2% sevoflurane, along with a continuous infusion of remifentanil at 0.8-1.2 mg/kg/min. Blood pressure was maintained above 65 mmHg with bolus phenylephrine and ephedrine.

One hour after the start of surgery, the potassium level rose from 4.6 to 5.65 mEq/L (Table 1), and 40 g of glucose with 10 units of insulin was administered. At the end of the surgery, the potassium level was 4.71 mEq/L. The operation lasted 129 minutes, during which the patient received a total of 1,600 mL of normal saline, with 100 mL urine output. The patient was subsequently diagnosed with postrenal failure due to urinary tract obstruction caused by bladder cancer, leading to the placement of a right nephrostomy.

**Table 1:** Atrial blood gas data in Case 1 GI infusion was initiated in the first hour of the total thyroidectomy. BE, base excess; Epi, epidural anesthesia; GA, general anesthesia; GI, glucose-insulin; Hb, hemoglobin; Lac, serum lactase; PNB, peripheral nerve block; Spi, spinal anesthesia

Surgery type	Total thyroidectomy				Transurethral resection of tumor	
Surgery time (h)	0	1	1.5	2	0	2
Anesthetic modality	GA				Spi	
pH	7.347	7.279	7.172	7.299	7.331	7.553
pCO_2 _(mmHg)	43.4	41	36.5	37.8	44.9	21.4
pO_2 _(mmHg)	162.1	158.3	186.1	159.3	16.9	76.5
HCO_3_ (mEq/L)	17.6	13.2	16.8	14.4	23.2	18.4
BE (mEq/L)	-8.1	-9.8	-11.7	-11.8	-2.7	-3.9
Hb (g/dL)	14.5	14	13.1	11.4	13.8	12.8
Na^+ ^(mEq/L)	136.7	135.2	135.7	132.2	135.7	132.4
K^+ ^(mEq/L)	4.6	5.65	4.95	4.71	4.6	4.22
Lac (mg/dL)	11.7	9.2	15	15	24.6	12.8

One month later, the patient was scheduled for transurethral resection of a bladder tumor under spinal anesthesia, using 11 mg of bupivacaine following a lumbar puncture. Loss of cold sensation at Th6 was confirmed, and blood pressure was maintained above 65 mmHg without any vasopressor. During surgery, the potassium level decreased from 4.6 to 4.22 mEq/L (Table 1). The operation time was 87 minutes, with a total of 900 mL of normal saline administered and unquantified urine output.

Case 2

A 72-year-old male (height: 163 cm; weight: 67 kg) with rheumatoid arthritis was scheduled for artificial joint replacement and tendon transplantation of his finger under a combination of general and regional anesthesia. His medical history included CKD grade 3a (sCr level: 1.1 mg/dL; eGFR: 51.02 mL/min/1.73m²), chronic obstructive pulmonary disease, atrial fibrillation, and permanent pacemaker implantation due to sick sinus syndrome.

In the operating room, general anesthesia was induced using 100 mg of propofol, 100 mg of fentanyl, and 40 mg of rocuronium. An endotracheal tube was inserted, and continuous arterial pressure was monitored via the radial artery. A right interscalene plexus nerve block was performed using 20 mL of 0.5% ropivacaine. Anesthesia was maintained with a combination of 35-50% oxygen, air, and 1.2% sevoflurane, along with a continuous infusion of remifentanil at 0.012 mg/kg/min and 20 mg/h of rocuronium. Blood pressure was maintained with bolus phenylephrine and ephedrine, keeping the mean arterial pressure above 65 mmHg. A tourniquet was used for 244 minutes.

Four hours after the start of the operation, the potassium level increased from 4.36 to 5.48 mEq/L (Table 2), prompting the administration of 20 g of glucose and 4 units of insulin. At the end of the surgery, the potassium level was 4.44 mEq/L. The operation lasted 337 minutes, during which the patient received a total of 1,200 mL of normal saline and had 400 mL of urine output.

**Table 2:** Arterial blood gas data in Case 2 GI infusion was initiated in the fifth hour of the artificial joint replacement and tendon transplantation of the finger. BE, base excess; Epi, epidural anesthesia; GA, general anesthesia; GI, glucose-insulin; Hb, hemoglobin; Lac, serum lactase; PNB, peripheral nerve block; Spi, spinal anesthesia

Surgery type	Artificial joint replacement and tendon transplantation of finger				Bilateral total knee arthroplasty		
Surgery time (h)	0	3	5	6	0	2	5
Anesthetic modality	GA+PNB				Epi+Spi		
pH	7.336	7.384	7.43	7.42	7.419	7.405	7.415
pCO_2 _(mmHg)	46.5	42.3	36.8	36.2	40	39.5	38.2
pO_2 _(mmHg)	257.7	161.4	158.4	161.8	40	97.9	96.5
HCO_3_ (mEq/L)	26	24.7	23.9	23	25.3	24.2	23.9
BE (mEq/L)	0.7	-0.4	-0.4	-1.5	0.8	-0.5	-0.6
Hb (g/dL)	11.8	11	10.9	10.5	12.4	11.6	11
Na^+ ^(mEq/L)	137.6	135.7	136.4	134.8	136.9	136.6	136.4
K^+ ^(mEq/L)	4.36	5.09	5.48	4.44	4.12	4	3.99
Lac (mg/dL)	9.6	17.6	19.3	23.6	11.2	8.4	16.3

Two years later, the patient underwent bilateral total knee arthroplasty. His medical history remained largely unchanged, with similar renal function (sCr level: 1.1 mg/dL; eGFR: 51.02 mL/min/1.73m²). Based on previous anesthesia records, regional anesthesia was used without general anesthesia. In the operating room, a lumbar epidural catheter was placed at the L1/L2 interspace, followed by spinal anesthesia with 0.5% tetracaine in 0.9% normal saline with 0.125% phenylephrine and 10 mg fentanyl at the L3-4 interspace. Continuous arterial pressure was monitored via the radial artery. After three hours, 1.5% lidocaine and 10 mg fentanyl were added via the epidural catheter. Blood pressure remained stable (mean arterial pressure: 65-75 mmHg), and the patient reported no pain with loss of cold sensation at Th6-8. A tourniquet was used for 218 minutes.

During this surgery, the potassium level decreased from 4.12 to 3.99 mEq/L (Table 2). The operation lasted 251 minutes, with a total of 1,750 mL of normal saline and 500 mL of urine output.

## Discussion

In our two cases, we observed an elevation in serum potassium levels under general anesthesia, while potassium levels remained stable under regional anesthesia alone. Few studies have explored the relationship between intraoperative hyperkalemia and regional anesthesia, and conflicting reports exist regarding the role of regional anesthesia in renal protection [4,5].

One possible mechanism for the elevation in serum potassium levels under general anesthesia is that mechanical positive pressure ventilation reduces venous return and cardiac output, thereby diminishing renal perfusion. This may contribute to renal injury and impair potassium excretion in the urine [6]. Additionally, mechanical hypoventilation can lead to respiratory acidosis, causing a shift of potassium from intracellular to extracellular compartments [7]; however, in our cases, the pCO2 levels did not increase. Previous animal studies have shown that volatile anesthesia reduces renal perfusion [8]. In contrast, regional anesthesia can block sympathetic nerve activity, thereby maintaining renal blood flow and mitigating postoperative renal inflammation. This may help preserve renal function and stabilize serum potassium levels [2].

Our study has several limitations. First, the intraoperative conditions were not identical between the general and regional anesthesia cases. Intraoperative renal function is influenced by numerous factors, including mean arterial pressure, fluid balance, blood loss, the type and duration of surgery, and preoperative medical history [9,10]. In our cases, only a few factors were comparable. Case 1 involved tumor resection, which could have elevated serum potassium levels due to tumor lysis, while Case 2 involved arthroplasty with a tourniquet, which also contributed to serum potassium elevation. These factors may be considered comparable in their potential to induce hyperkalemia under general and regional anesthesia. The mean blood pressure was consistently maintained above 65 mmHg, which likely minimized the effect of hypotension on renal function [9]. Second, in both cases, after experiencing hyperkalemia during surgery under general anesthesia, the patients were managed with regional anesthesia. This sequence of anesthesia management may have influenced the outcome under regional anesthesia.

## Conclusions

Our cases suggest that regional anesthesia may have the potential to prevent intraoperative hyperkalemia in patients with CKD. While most surgeries are typically performed under general anesthesia without a significant increase in serum potassium in patients with CKD, our two cases may represent an exception. Therefore, the interpretation of these results should be approached with caution. However, the accumulation of similar cases is essential to enhance our understanding of the effects of regional anesthesia on perioperative renal function. Further studies are needed to elucidate the impact of regional anesthesia on intraoperative renal function.
